# Antiviral Activity of *Rosa damascena* Mill. and *Rosa alba* L. Essential Oils against the Multiplication of Herpes Simplex Virus Type 1 Strains Sensitive and Resistant to Acyclovir

**DOI:** 10.3390/biology10080746

**Published:** 2021-08-04

**Authors:** Neli Vilhelmova-Ilieva, Ana Dobreva, Rositsa Doynovska, Dimo Krastev, Milka Mileva

**Affiliations:** 1 The Stephan Angeloff Institute of Microbiology, Bulgarian Academy of Sciences, 26 Acad. G. Bonchev Str., 1113 Sofia, Bulgaria; nelivili@gmail.com; 2 Institute for Roses and Aromatic Plants, 49 Osvobojdenie Blvd, 6100 Kazanlak, Bulgaria; anadobreva@abv.bg; 3 Faculty of Public Health, Health Care and Sport, South-West University “Neofit Rilski”, 66 Ivan Mihailov Str., 2700 Blagoevgrad, Bulgaria; doynovska@swu.bg (R.D.); dimo_krustev@mail.bg (D.K.); 4 Medical College “Jordanka Filaretova”, Medical University of Sofia, 3 Jordanka Filaretova Str., 1606 Sofia, Bulgaria

**Keywords:** herpes simplex virus type 1 (HSV-1), acyclovir-resistant (R-100) strain, acyclovir, *Rosa damascena* Mill. and *Rosa alba* L. oil, combined effect

## Abstract

**Simple Summary:**

Herpes simplex virus type 1 (HSV) is a coated DNA virus of the Herpesviridae family. It causes painful infections of the mouth, throat, face, eyes, central nervous system, as well as infections of the anal–genital area. The specific drugs for chemotherapy of HSV have been based on nucleoside analogues, with acyclovir (ACV) being the most widely used. The most serious problem in the application of nucleoside analogues is the rapid formation of resistant mutants, which also often leads to treatment failure. The search for new therapeutic alternatives for the treatment of HSV is necessary for the successful control of diseases caused by herpes infection. Rose essential oils are widely used in alternative medicine due to their many proven benefits for human health. In the treatment of bacterial and viral infections, they reduce the chance of developing resistance. In this study, we investigated the effects of the Bulgarian *Rosa damascena* Mill. and *Rosa alba* L. essential oils on the viral reproduction of susceptible (Victoria strain) and acyclovir-resistant (R-100) strains individually and in combination with acyclovir. When the rose oils were added after the virus entered the cell, co-administered with ACV at a concentration four times lower than the IC50, they contributed to a significant reduction in viral yield by more than 20% of the expected inhibition of viral replication in the Victoria strain and more than 10% of the previously presumptive inhibition in the R-100 strain.

**Abstract:**

Background: The specific chemotherapeutics against herpes simplex virus type 1 (HSV) are nucleoside analogues such as acyclovir (ACV), but the most important problem is the formation of resistant mutants. The search for new therapeutic alternatives leads us to the purpose of investigating the effects of *Rosa damascena* Mill. and *Rosa alba* L. essential oils on the viral reproduction of susceptible (Victoria) and acyclovir-resistant (R-100) strains of HSV-1 replication *in vitro*, individually and in combination with acyclovir. Methods: Cytopathic effect inhibition test was used for assessment of antiviral activity of the oils, and the three-dimensional model of Prichard and Shipman was applied to evaluate the combined effect of oils with ACV on HSV-1 replication. Results: Both oils do not affect the replication of viral strains; they are able to influence only viral adsorption and extracellular virions and protect healthy cells from subsequent infection. In combination with lower doses of acyclovir, both oils demonstrate a significant synergistic effect on the replication of HSV-1, which is more contagious than the Victoria strain. Conclusions: The nonspecific mechanism of the reduction in viral reproduction caused by rose oils and the synergistic effect of their co-administration with the lower doses of specific inhibitor ACV makes them suitable therapeutics for overcoming viral resistance to HSV-1 infections.

## 1. Introduction

Last year showed humanity a significant example of the dangers and seriousness of the prevention, treatment and control of viral diseases via the COVID 19 pandemic. Other dangerous infections that have also spread around the world, described as early as the time of Hippocrates as dangerous skin lesions, are the infections caused by the herpes simplex virus type 1 and 2 (HSV-1 and HSV-2) [1]. According to Roizman et Knipe (2001), HSV is one of the most difficult viruses to control and has plagued humankind for thousands of years [2].

Herpes simplex virus is a coated DNA virus of the Herpesviridae family. It causes painful infections of the mouth, throat, face, eyes, skin and the mucous membrane, the central nervous system, as well as infections of the anal–genital area [1,2,3]. The specific drugs for chemotherapy of HSV have been developed on the basis of nucleoside analogues, among them acyclovir (ACV) being the most widely used. In addition, the most serious problem in the application of nucleoside analogues is the rapid formation of resistant mutants, which often leads to treatment failure [4,5]. The search for new therapeutic alternatives for the treatment of HSV is necessary for the successful control of diseases caused by herpes infection [1,4].

For hundreds of years, people used a variety of plants for the treatment of various virus diseases. Many plant extracts show antiherpes virus activity [1,4,5].

Essential oils derived from the distillation of some aromatic plants (e.g., *Melaleuca alternifolia*, *Mentha piperita* and *Thymus vulgaris*), as well as isolated single essential oil components (e.g., eugenol, nerol, linalool, carvacrol, thymol), display antiviral properties against enveloped viruses, including herpes viruses. Research conducted recently has shown that essential oils can play a significant supporting role in the prevention and treatment of herpes infections. They are a “rich cocktail of ingredients” [1,5,6,7], including monoterpenes, sesquiterpenes, phenylpropane derivatives, alcohols, aldehydes, ketones, esters, organic acids and others with rich biological properties. Two ways of introducing oils into the body are most effective: (i) inhalation—through the respiratory tract, it is the basis of aromatherapy and (ii) topically through the skin such as creams, compresses and baths. Due to their lipophilic nature, essential oils have excellent penetration through the skin, mainly through hair follicles and sebaceous and sweat glands, which means that in mature and hard-to-reach areas, they can provide very good bioavailability and therapeutic effect [7,8,9,10,11]. In the scientific literature, there have been several attempts to discover synergistic combinations of antiviral compounds, such as ACV, and some main ingredients of essential oils (geraniol) as a therapeutic adjuvant [12]. The advantages of synergistic combinations of antiviral compounds are reduced doses of potentially toxic compounds, side effects, reduced probability for the emergence of drug-resistant viruses and increased antiviral potency [11,12,13].

The essential oils of Bulgarian *Rosa damascena* Mill. and *Rosa alba* L. have been paid attention to not only for the beauty and fragrance of rose flowers [14] but because of their healing effect. Both roses have a place in Bulgarian folklore and traditional medicine. From a scientific point of view, empirical knowledge from traditional medicine is an important aspect of future *in vitro* and *in vivo* studies. In our previous studies, we discovered that the essential oils of *Rosa damascena* Mill. and *Rosa alba* L. do not show or have low genotoxic activity. The data about their cytotoxic/anticytotoxic, mutagenic/antimutagenic and genotoxic/antigenotoxic potentials are of particular importance for the protection of the cell genome from damage induced by various factors. The results reported showed that these oils are cytotoxically and genotoxically safe products [15,16,17].

The aim of this study was to investigate the effects of the Bulgarian *Rosa damascena* Mill. and *Rosa alba* L. essential oils on the replication of susceptible (Victoria strain) and acyclovir-resistant (R-100) strains of HSV-1, individually and in combination with acyclovir.

## 2. Materials and Methods

### 2.1. Plants’ Material and Distillation of Essential Oils

We used as raw material the fresh petals of *Rosa alba* L. and *Rosa damascena* Mill. from a plantation in the experimental field of the Institute of Rose and Essential Oil Plants in Kazanluk. The plant material was gathered at the time of the collection campaign in the morning of May/Jun 2020, before sunrise (6–8 a.m.), in a phase of flowering semi-blooming-blooming flowers. Rose oil was distilled immediately by water–steam distillation on a semi-industrial processing line at the Institute. Process parameters of the distillation were as follows: raw material for a charge, 10 kg; hydro module 1:4, rate of 8–10% and call duration of 150 min. The essential oil was dried with sodium sulphate, filtered and stored appropriately.

### 2.2. Chromatographic Conditions

The chromatographic analysis of essential oils was performed using Agilent 7890A/5975 GC MS system, equipped with an HP-5 apolar column (60 m × 0.25 mm × 0.25 m). As a carrier gas, helium with a constant flow rate of 1 mL/min was used. The splitless injection of a 1 mL sample was performed. The parameters of the temperature programme were indicated as described in the international ISO 9842 standard [18]. The identification of compounds was performed by comparison of their relative retention indices and mass spectra with those of pure substances. Mass spectra were also compared with those of the National Institute of Standards and Technology (NIST) library database.

### 2.3. Cells

Madin-Darby bovine kidney (MDBK) cells were obtained from the National Bank for Industrial Microorganisms and Cell Cultures, Sofia. The cell lines were grown in a DMEM medium containing 10 mM HEPES buffer (Merck, Darmstadt, Germany), 10% fetal bovine serum (Gibco BRL, Waltham, MA, USA) and antibiotics (penicillin 100 IU/mL, streptomycin 100 μg/mL). The cell culture was incubated in a CO_2_ incubator (HERA cell 150, Heraeus, Hanau, Germany) at 37 °C/5% CO_2_.

### 2.4. Viruses

Herpes simplex virus type 1 (HSV-1), Victoria strain, was obtained by Prof. S. Dundarov, National Center of Infectious and Parasitic Diseases, Sofia. MDBK cell culture in DMEM (Gibco BRL, Paisley, Scotland, UK) containing 0.5% fetal bovine serum (Gibco BRL, Scotland, UK) was used to replicate the virus. The infectious viral titer of the viral stock is 10^8.0^ CCID_50_/mL.

Herpes simplex virus type 1, Victoria strain (HSV-1), was received from Prof. S. Dundarov, National Center of Infectious and Parasitic Diseases, Sofia. The virus was replicated in monolayer MDBK cells in a maintenance solution DMEM (Gibco BRL, Paisley, Scotland, UK) plus 0.5% fetal bovine serum (Gibco BRL, Scotland, UK). The infectious titer of the stock virus was 10^8.0^ CCID_50_/mL.

Acyclovir-resistant herpes simplex virus type 1 (strain R-100), characterised by a mutation in the gene encoding the enzyme, TK, was used and its substrate specificity was altered (TK^a^, HSV-1) (provided by the Laboratory of Virology, Sofia University “St. Kliment Ohridski”). The infectious titer of the viral stock is 10^6.0^ CCID_50_/mL.

### 2.5. Cytotoxicity Assay

A confluent monolayer cell culture in 96-well plates (Costar^®^, Corning Inc., Kennebunk, ME, USA) was treated with 0.1 mL/well containing a maintenance medium with falling concentrations of the tested oils. Cells were incubated at 37 °C and 5% CO_2_ for 48 h. After microscopic evaluation, the medium containing the test products was removed, and 150 mL/well of PBS was added over a short time and removed together with the residues of the solutions of the substances. To each sample was added 0.100 mL/well containing a neutral red (NR) dye (a stock solution with a concentration of 0.4% diluted 1:80 in support medium). The cells were incubated for 3 h at 37 °C/5% CO_2_. The dye was then removed, and the cells were washed with PBS, 0.150 mL/well. Desorbed solution (1% glacial acetic acid and 49% ethanol in distilled water), 0.15 mL/well, was added to recover and dilute the NR absorbed by the viable cells. The optical density (OD) of each sample was read at 540 nm in a microplate reader (Biotek Organon, West Chester, PA, USA). The 50% cytotoxic concentration (CC_50_) was defined as the oil’s concentration that reduced the cell viability by 50% when compared with the untreated control. The experiments were carried out in triplicate with three parallels per sample.

The maximum tolerable concentration (MTC) of the oils was also determined. This is the concentration at which the oils do not affect the cell monolayer, and, in the sample, it resembles the cells in the control sample (untreated with the oils).

### 2.6. Antiviral Activity Assay

The antiviral activity of the oils was determined using a cytopathic effect inhibition (CPE) test. The confluent cell monolayer in 96-well plates was infected with 100 cell culture infectious dose 50% (CCID_50_) in 0.1 mL (Victoria or R-100 strain). After 60 min of virus adsorption, the non-adsorbed virus was removed, and extracts were added in various concentrations. After that, cells were incubated for 48 h at 37 °C. A neutral red uptake assay was used to account for the cytopathic effect, and the percentage of CPE inhibition for each sample was calculated by the following formula:

% CPE inhibition = [OD_test sample_ − OD_control infected sample_]/[OD_toxicity control_ − OD_control infected sample_] × 100

where OD_test sample_ is the mean value of the ODs of the wells inoculated with virus and treated with the test sample in the respective concentration tested, OD_control infected sample_ is the mean value of the ODs of the untreated infectious samples (with no oil in the medium) and OD_toxicity control_ is the mean value of the ODs of the wells not inoculated with virus but treated with the corresponding concentration of the test sample. A 50% inhibitory concentration (IC_50_) of the oils was determined as the concentration of test products that inhibits 50% of viral replication compared with the control infected sample. The selectivity index (SI) was calculated from the ratio CC_50_/IC_50._

### 2.7. Virucidal Assay

A contact sample was prepared in the proportion 1:1 containing HSV-1 (Victoria strain) (10^4^ CCID_50_), and the test oil was pre-prepared in its 2MTC so that when the virus was added, the oil solution was diluted to twice the working concentration of MTC. At the same time, a control sample was prepared to contain only the virus in the same concentration, diluted in a maintenance medium. The prepared sample and control were, thus, incubated at room temperature for various time intervals (5, 15, 30, 60, 90 and 120 min). The residual infectious virus content of each sample was determined by the final dilution method, and the reduction in Δlgs of the virus infectivity compared with the viral controls was determined.

### 2.8. Virus Attachment Assay 

A total of 24-well cell culture plates containing a monolayer of MDBK cells were pre-chilled at 4 °C, inoculated with 10^4^ CCID_50_ of HSV-1 (Victoria strain) for adsorption at 4 °C and treated simultaneously with MTC of the oils applied separately. At various time intervals (15, 30, 45, and 60 min), cells were washed with PBS in individual samples in order to remove both the compound and the unattached virus, then overlaid with maintenance medium and incubated at 37 °C for 24 h. Under the same conditions and for the same time intervals, control samples containing the same amount of virus but not treated with the test substance (i.e., they contained only a maintenance medium) were prepared simultaneously. After triple freezing and thawing the samples, the infectious viral titer of each sample was determined by the endpoint dilution method. Viral titers of the samples were compared with the viral titer of the virus control for each time interval, and Δlgs of virus yield reduction were determined. Each sample was prepared in triplicate.

### 2.9. Pretreatment of MDBK Cells

Monolayers of MDBK cells pre-grown into 24-well cell culture plates (CELLSTAR, Greiner Bio-One GmbH, Kremsmünster, Austria) (2 × 10^6^ cells per well) were treated for 5, 15, 30, 60, 90 and 120 min at a concentration of MTC of the rose oils in the maintenance medium (1 mL per well). The tested products were then removed, and the cells were washed with phosphate-buffered saline (PBS) and inoculated with HSV-1 (Victoria strain) (1000 CCID_50_ in 1 mL per well). After 60 min of absorption, the non-absorbed virus was removed, and the cells were covered with a maintenance medium. The culture plates were incubated at 37 °C for 24 h and, after triple freezing and thawing, the infectious viral titers were determined by the endpoint dilution method. Δlgs of virus yield reduction were evaluated compared with the control infected sample (untreated by compounds).

### 2.10. Combination Effect Analysis

The combined effects between oils and acyclovir were determined using a three-dimensional model developed by Pritchard and Shipman [19], and the effect was assessed using the computer programme MacSynergy™ II [20]. The programme uses confidence intervals for the statistical evaluation of drug interactions. It presents the effect as a horizontal plane at 0% inhibition. The peak above this surface represents a higher value than the expected antiviral activity (i.e., synergy), while any depression below the zero surface shows antagonism [19]. The programme also calculates the volume of synergy and/or antagonism in μg/mL^2^%: (I) values of synergy or antagonism below 25 μg/mL^2^% at 95% confidence intervals should be considered insignificant, (II) values between 25 μg/mL^2^% and 50 μg/mL^2^% should be considered weak but with a significant amount of synergy, (III) values between 50 μg/mL^2^% and 100 μg/mL^2^% indicate moderate synergy or antagonism and (IV) values above 100 μg/mL^2^% show strong synergy [20]. Combination antiviral effects were evaluated through the FFU reduction method in which the microscopically registered viral foci formed in the cell monolayer were counted. The experiments were performed four times, and each sample was repeated four times in each trial.

### 2.11. Statistical Analysis

Data on cytotoxicity and antiviral effects were analysed statistically. The values of CC_50_ and IC_50_ were presented as mean ± SD. The significance between the difference in the cytotoxicity values of rose oils and ACV was performed through the student’s *t*-test in which *p*-values of <0.05 were regarded as significant.

## 3. Results

### 3.1. Chromatographic Profile of the Essential Oils

**Figure 1:**
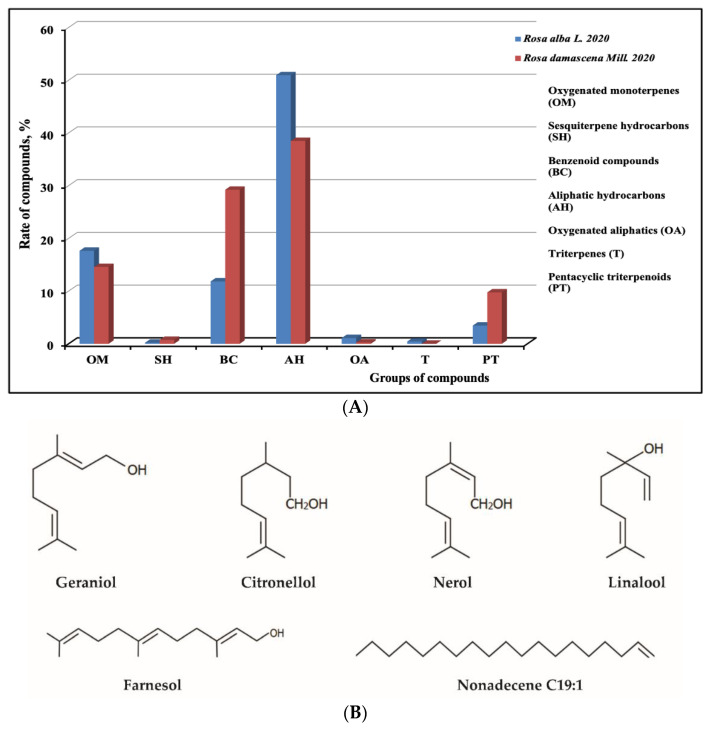
Main groups of compounds containing essential oils of Bulgarian *Rosa alba* L., and *Rosa damascena* Mill (**A**). Chemical structure of main ingredients of rose oils (**B**).

Chromatographic composition of the essential oils of *R. alba* L. and *R. damacena* Mill. was studied by GC/MC, and the main classes of compounds are presented in Figure 1. In accordance with Nedkov et al. (2009) [21], the chromatographic profile is typical for representatives of the Rosa family (Table S1 of Supplementary Material). The main groups of compounds are hydrocarbons with a high molecular weight (C15 C31), such as nonadecane and heneicosane, followed by monoterpenes (geraniol, nerol, citronellol) and then followed by trace amounts of phenylpropanoids such as eugenol and methyeugenol and terpenoids such as neral and geranial. Rose oils comprise a complex mixture of ingredients, and their biological activities can be attributed to their different constituents [22].

### 3.2. Study on Antiviral Properties

#### 3.2.1. Determination of Cytotoxic Concentration

For the correct performance of the antiviral experiments, the cytotoxic concentration of the tested products, which affects 50% of the cell monolayer of MDBK cells (CC_50_), had to be determined first. According to the values of this concentration, the oils of both roses demonstrated cytotoxicity about twice as low as that of acyclovir (Table 1). When comparing the two oils among themselves, that of *R. damascena* showed slightly greater toxicity (CC_50_ = 530 µg/mL) to MDBK cells compared with that of *R. alba* L. oil (CC_50_ = 570 µg/mL). The maximum tolerable concentration of the oils (MTC) at which they do not affect the cell monolayer was also determined. This oil concentration was used in most antiviral experiments, and the same value (10 µg/mL) was found for both oils (Table 1).

#### 3.2.2. Effect of Essential Oils on the Replication of HSV-1

**Figure 2:**
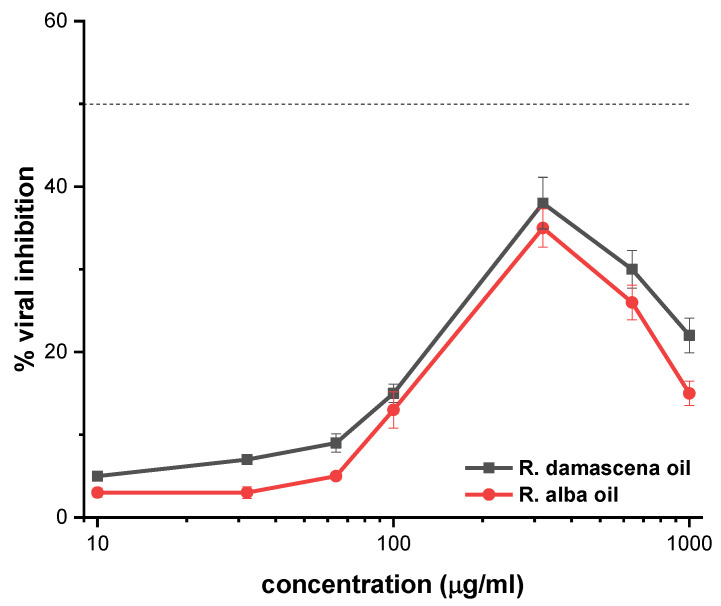
Antiviral activity of *Rosa damascena* Mill. and *Rosa alba* L. essential oils on the one replication of HSV-1. The experimental conditions are described in Section 2.6.

Rose oils did not significantly inhibit the replication of the HSV-1 strains tested. The established inhibition did not exceed 40% (Figure 2) and, therefore, their selective indices cannot be determined because they are defined as a CC_50_/IC_50_ ratio (Table 1).

#### 3.2.3. Effect of Essential Oils on the Viability of Virions

The inhibitory effect of rose oils on the viability of HSV-1 virions was also evaluated (Table 2). Both oils showed their effect at 15 min of exposure by lowering the viral titer by Δlg = 1.75. As the exposure of time increased, viral infectivity decreased. In the studied time intervals between 30–90 min, the oil of *R. alba* L. had a slightly more pronounced effect, but at the last time interval of 120 min, the extent of suppression was again equalised (Δlg = 3.0).

#### 3.2.4. Effect of Essential Oils on the Viral Adsorption

The influence of rose oils on the stage of viral adsorption is presented in Table 3. At 15 min of exposure, the effect of both oils was weak. The attachment of viral particles to sensitive MDBK cells was significantly inhibited after 30 min and was time-dependent—with increasing exposure time, the number of attached viruses decreased. The effect caused by *R. damascena* Mill. oil was more pronounced, reducing the viral titer by Δlg = 4.0 at 60 min of exposition.

#### 3.2.5. Protective Effect in Pretreatment of Healthy MDBK Cells

We also studied the protective effect of rose oils on the membrane of MDBK cells before they came into contact with the virus (Table 4). The results show that the oils had a positive effect on the cell membrane and reduced the subsequent attachment of viral particles. At 15 min of cell pretreatment, the effect was weak and at the next time interval studied (30 min), it was clearly manifested. This effect depended on the treatment time and was more pronounced in *R. damascena* Mill. oil, with the strongest protective effect observed at 120 min of incubation (Δlg = 4.25 reduction of viral titer).

The rose oils we studied did not show a significant disruption of the viral replication cycle but clearly inhibited extracellular virions, their attachment and penetration into the cell. Based on these results, we decided to investigate their effect on viral reproduction applied in combination with ACV. The effect was determined in both acyclovir-sensitive (Victoria) and resistant (R-100) strains.

#### 3.2.6. Combined Effect between *R. damascenа* Mill. Oil and ACV versus Victoria Strain Replication

The character of the partners’ relationships in the combinations was assessed using the computer programme MacSynergy™ II at a 95% confidence interval. Acyclovir was applied in a concentration of IC_50_ and multiples of it and rose oils were in their MTC and multiples of it.

**Figure 3:**
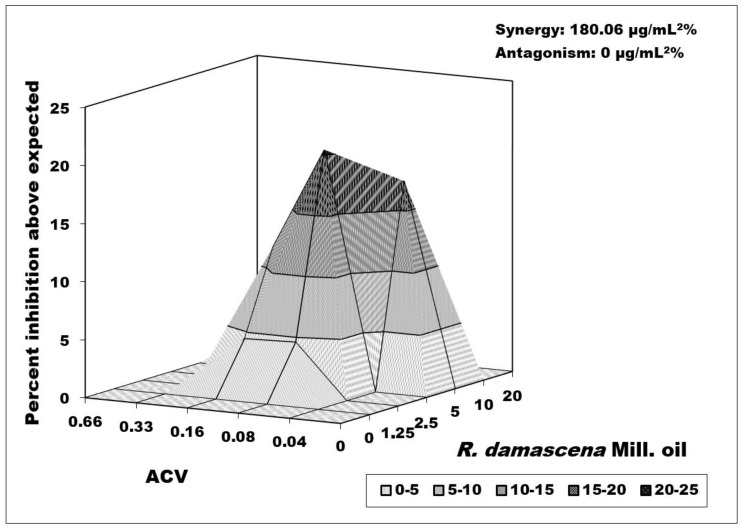
Combined effect of *Rosa damascena* Mill. essential oil and acyclovir on HSV-1 replication (Victoria strain). Data processing is described in detail in Section 2.10.

The combined effect between *R. damascenа* Mill. oil and ACV versus the Victoria strain replication showed a strong synergistic effect of 180.06 μg/mL^2^%. The zone of synergism covered the lower concentrations of ACV (IC_50_/2—IC_50_/8) and the higher doses in *R. damascenа* Mill. oil (2MTC—MTC/2). We had the strongest peak of the synergistic effect at concentrations of 0.16 µg/mL for ACV and 10 µg/mL for rose oil (Figure 3).

#### 3.2.7. Combined Effect between *R. alba* L. Oil and ACV versus Victoria Strain Replication

**Figure 4:**
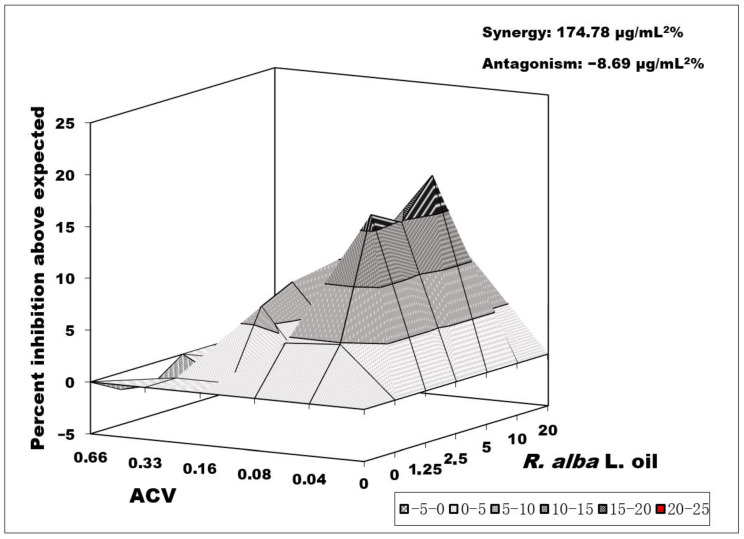
Combined effect of *Rosa alba* L. oil and acyclovir on HSV-1 replication (Victoria strain). Data processing is described in detail in Section 2.10.

The second combination studied on the replication of Victoria strain was that of ACV and *Rosa alba* L. oil, and it also showed a marked synergistic effect (174.78 μg/mL^2^%) that was more significant at therapeutic doses of ACV equal or lower than its IC_50_. *R. alba* L. exhibited its synergistic effect at MTC/2 concentrations and higher. The synergistic peak of the combination was at concentrations of 0.08 µg/mL of ACV and 20 µg/mL of the tested oil. A negligible antagonistic effect (−8.69) was observed at high ACV concentrations and low concentrations of *Rosa alba* L. oil (Figure 4).

From the values of the synergistic effect of the two studied combinations on the replication of the Victoria strain, it can be assumed that the obtained effect is clearly distinct and would be significant in an *in vivo* study.

#### 3.2.8. Combined Effect between *Rosa damascena* Mill. Oil and ACV versus R-100 Strain Replication

**Figure 5:**
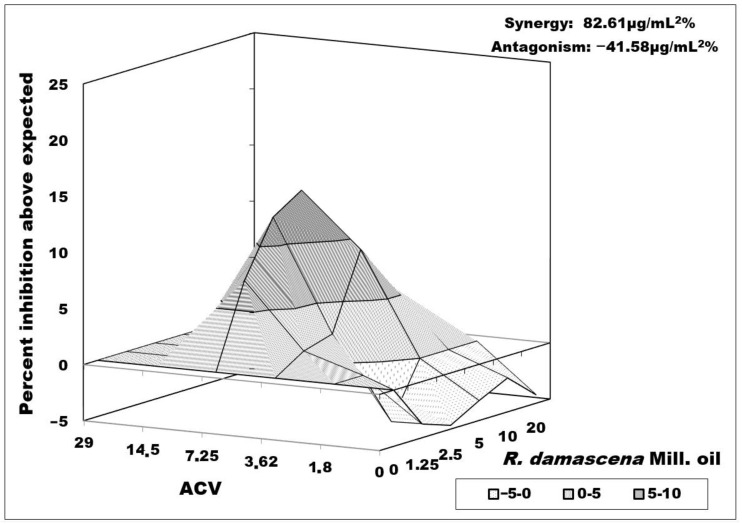
Combined effect of *Rosa damascena* Mill. oil and acyclovir on HSV-1 replication (R-100 strain—resistant to ACV). Data processing is described in detail in Section 2.10.

Both combinations studied on the replication of the R-100 strain showed moderate synergism. A synergistic effect of 82.61 μg/mL^2^% was observed with the simultaneous application of ACV and *Rosa damascena* Mill. oil (Figure 5). The area of synergism was at ACV IC_50_/2 and IC_50_/4 concentrations and oil concentrations equal and higher than MTC/4. A synergistic peak was observed when combining ACV at 3.62 µg/mL and *Rosa damascena* Mill. oil 5 µg/mL. Some antagonism (−41.58 μg/mL^2^%) was also observed when applying ACV in concentrations of IC_50_/8, and at 2IC_50_, the effect was additive with slight antagonism.

#### 3.2.9. Combined Effect between *Rosa alba* L. Oil and ACV versus R-100 Strain—Resistant to ACV Replication

**Figure 6:**
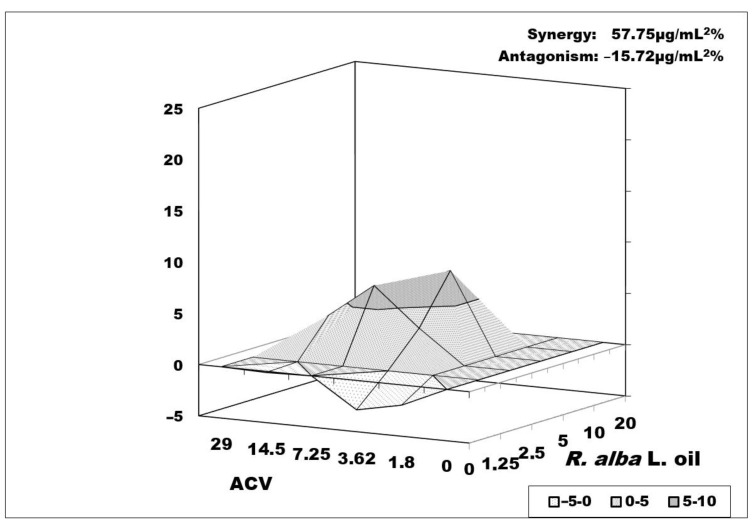
Combined effect of *R. alba* and acyclovir on HSV-1 replication (R-100 strain—resistant to ACV). Data processing is described in detail in Section 2.10.

The combination of *R. alba* L. oil and ACV (Figure 6) had a similar character but with a weaker synergistic effect (57.75 μg/mL^2^%). The zone of synergism that was formed was the same as the combination with the oil of *R. damascena* Mill., but with the formation of two not very high peaks of synergism in ACV (7.25 µg/mL) and *R. alba* L. oil (5 µg/mL), and in ACV (3.62 µg/mL) + oil (10 µg/mL). Again, at high concentrations of ACV, a weak antagonism (15.72 μg/mL^2^%) was observed mixed with zones of an additive effect. Although the combinations of test oils with ACV versus replication of the resistant strain had moderate synergism, they could be used to reduce higher therapeutic doses of ACV.

## 4. Discussion

As a rule, effective antiviral drugs can affect one or more stages of viral reproduction on specific or nonspecific mechanisms. The antiherpes virus strategy of essential oils is either to directly inactivate the extracellular virions (virucidal effect) or to inhibit the different steps of HSV replication as virus attachment, penetration and intracellular targeting of the virions production [23].

In the scientific literature, there is insufficient data on the antiviral activity of essential oils from *Rosa alba* L. and *Rosa damascena* Mill. Figure 1A,B shows the chromatographic profiles of both oils. As it can be seen, they are a mixture of many biologically active substances that belong to the groups of oxidised monoterpenes, benzoid components, oxidised aliphatics and pentacyclic terpenoids. In the literature, there is much information about the biological activities of the components included in this composition [3,11,12,13,14,16,17,18]. The most important ingredients in rose oils are nerol, citronellal, citronellol, geraniol, eugenol and others. Some of these substances have proven antiviral activity affecting certain stages of viral replication [24,25]. The content of essential oils with antiherpes activity isolated from other plant species is similar. Many of these essential oils exhibit virucidal activity by binding non-specifically to HSV-1 particles [24,25,26,27,28,29].

To analyse the mode of antiviral action of Bulgarian rose essential oils, we studied the oils using standard experimental protocols recommended by Cos et al. (2006) [30]. Victoria strain of HSV-1 was treated with essential oils at different stages of virus replication (Table 1, Table 2, Table 3 and Table 4).

In the infected body, in the conditions of developing herpesvirus infection, therapeutics acting in the pre-viral stages are of particular importance because it helps reduce the ability of viral particles to pass from cell to cell and helps reduce the virulence of the virus.

It is known that essential oils have a lipophilic nature [11] that helps to bind them to the lipids of the herpes virus envelope. This leads to its inactivation and disruption of the process of its attachment to the host cell. This effect may be due to both virion inactivation and binding to lipids from the cell membrane, making it difficult for the virus to attach to and enter the cell. The antiherpes virus activity of essential oils and their main compounds could be connected as well with their ability to act as a natural antioxidant [31], modulating the cell’s redox balance as radical scavengers [32,33,34,35,36].

Despite the weak individual effect of rose oils, in combination after the virus enters the cell, co-administered with ACV, they contribute to a reduction in the viral yield. They successfully complement the action of ACV, preventing the passage of newly obtained viral particles from cell to cell and inactivating extracellular virions. Unfortunately, the body infected with the herpes virus often suffers from very severe symptoms during the infection. In this case, in addition to the specific suppression of viral replication, symptomatic treatment is required to alleviate suffering. Of course, the specific role at this stage is played by the specific antiviral, which suppresses a certain stage of viral replication. In the context of developing an infection, therapeutic approaches operating in the pre-viral stages are of particular importance because they are a serious barrier for viral particles to pass from cell to cell, which also helps to reduce the virulence of the herpes virus.

Of particular interest are the results obtained from the combined effects of the two essential oils with acyclovir on viral reproduction. The result showed that in the most pronounced synergism against the HSV-1 Victoria strain between *Rosa damascena* Mill. oil and acyclovir, ACV was applied at a concentration eight times lower than the IC_50_, providing us with a serious reason to hope that *in vivo* conditions, it will also be highly effective (Figure 3). Moreover, even higher antagonism was observed at higher ACV concentrations. Although the combinations of tested oils with ACV versus replication of the resistant strain had moderate synergism, they could be used to reduce high therapeutic doses of ACV, too.

These findings suggest that rose essential oils may be used as a typical prophylactic and/or supplementary therapeutic agent for herpes infections. However, further studies are required to illuminate the active constituents of this extract, which may be useful in developing new antiherpes drugs.

## 5. Conclusions

The different mechanisms by which they affect the reproduction of HSV-1 and the synergistic effect demonstrated when co-administered with ACV make the essential oils of Bulgarian *Rosa damascena* Mill. and *Rosa alba* L. potential therapeutics for herpes infections. They could be successfully applied in practice in combination with ACV and would lead to a reduction in drug resistance and faster recovery of the body during herpes infection.

## Figures and Tables

**Table 1:** Cytotoxicity (on MDBK cells) and anti-HSV-1 activity of *Rosa damascena* Mill. and *Rosa alba* L. essential oils.

Compound Tested	Cytotoxicity (µg/mL)		Antiviral Activity			
			Victoria Strain		R-100 Strain	
	CC_50_	MTC	IC_50_ (µg/mL)	SI	IC_50_ (µg/mL)	SI
*R. damascena* Mill. oil	530.0 ± 5.25 ***	10.0	-	-	-	-
*R. alba* L. oil	570.0 ± 5.83 ***	10.0	-	-	-	-
ACV	291.0 ± 9.4	-	0.33 ± 0.03	881.8	11.6 ± 1.2	25.3

*** *p* < 0.0001 when comparing each of the compounds tested with ACV; student’s *t*-test. CC_50_, 50% cytotoxic concentration; MTC, maximum tolerable concentration; IC_50_, 50% inhibitory concentration; SI, selectivity index. The experimental conditions are described in Section 2.5.

**Table 2:** Virucidal activity against HSV-1 (Victoria strain) virions of *Rosa damascena* Mill. and *Rosa alba* L. essential oils.

Compounds	Δlg of Infectivity Reduction					
	5 min	15 min	30 min	60 min	90 min	120 min
*R. damascena* Mill. oil	1.0	1.75	1.75	2.0	2.25	3.0
*R. alba* L. oil	1.0	1.75	2.0	2.25	2.5	3.0

The experimental conditions are described in Section 2.7.

**Table 3:** Effect on the adsorption of HSV-1 (Victoria strain) of *Rosa damascena* Mill. and *Rosa alba* L. essential oils.

Compound	Δlg of Virus Yield Reduction			
	15 min	30 min	45 min	60 min
*R. damascena* Mill. oil	1.5	2.0	2.75	4.0
*R. alba* L. oil	1.5	1.75	2.5	3.5

The experimental conditions are described in Section 2.8.

**Table 4:** Protective effect in pretreatment of healthy cells of *Rosa damascena* Mill. and *Rosa alba* L. essential oils.

Compound	Δlg of Virus Yield Reduction					
	5 min	15 min	30 min	60 min	90 min	1120 min
*R. damascena* Mill. oil	1.0	1.5	2.75	2.75	3.25	4.25
*R. alba* L. oil	1.0	1.25	2.0	2.5	3.50	3.75

The experimental conditions are described in Section 2.9.

## Data Availability

Data is contained within the article or Supplementary Material.

## Supplementary Materials

The following are available online at https://www.mdpi.com/article/10.3390/biology10080746/s1, Table S1: chromatographic profile of essential oils.
