# Supplementary material for: Antiviral Activity of Rosa damascena Mill. and Rosa alba L. Essential Oils against the Multiplication of Herpes Simplex Virus Type 1 Strains Sensitive and Resistant to Acyclovir
Source: Biology (Basel). 2021 Aug 4;10(8):746. doi: 10.3390/biology10080746 (PMC8389625; doi:10.3390/biology10080746)
Supplement: Supplementary file 1 [file biology-10-00746-s001.zip › biology-1291558-supplementary.pdf]

Table S1. Chemical composition of the *R. damasena* Mill. and *R. alba* L. essential oils.

The chromatographic analysis of essential oils was performed using Agilent 7890A/5975 GC MS system, equipped with an HP-5 apolar column (60 m x 0.25 mm x 0.25 m). As a carrier gas helium with a constant flow rate of 1 mL/min was used. The splitless injection of a 1 mL sample was performed. The parameters of the temperature program were as indicated in the standard described in the international standard ISO 9842 [19]. The identification of compounds was performed by comparison of their relative retention indices and mass spectra with those of pure substances. Mass s spectra also compared with these of the National Institute of Standards and Technology (NIST) library database.

Compounds					Essential oils	
					Rosa alba L.	*Rosa damascena* Mill.
**№**	**Name**	**Chemical groups**	**RT**	**RI**	**% of TIC**	
1	Linalool	OM	14.86	1097.2	0.75	nd
2	cis-Rose oxide	HM	15.33	1106.3	nd	0.26
3	Phenethyl alcohol	BC	15.40	1110.3	0.04	0.10
4	trans-Rose oxide	HM	15.77	1124.2	nd	nd
5	α-Terpineol	OM	17.65	1187.3	0.16	nd
6	β-Citronellol	OM	18.65	1225.5	23.24	26.56
7	cis-Geraniol	OM	18.70	1229.0	3.38	5.18
8	trans-Geraniol	OM	19.39	1250.4	11.62	15.33
9	Citronellyl acetate	OM	22.09	1350.5	nd	0.58
10	Geranyl acetate	OM	22.88	1380.0	0.06	0.78
11	β-Elemene	SH	23.07	1390.2	0.40	0.45

12	Methyleugenol	BC	23.39	1404.9	nd	0.05
13	β-Caryophyllene	SH	23.76	1419.1	3.91	1.21
14	β-Cubebene	SH	23.82	1424.1	nd	nd
15	β-Copaene	SH	24.03	1432.6	0.16	0.13
16	α-Guaiene	SH	24.24	1439.1	nd	0.52
17	α-Humulene (α-Caryophyllene)	SH	24.65	1454.3	0.24	0.31
18	γ-Muurolene	SH	25.19	1479.9	nd	0.08
19	Germacrene D	SH	25.34	1485.2	0.07	0.22
20	n-Pentadecane	AH	25.69	1500.2	0.95	0.34
21	α-Bulnesene	SH	25.93	1510.4	nd	0.29
22	δ-Cadinene	SH	26.34	1523.7	0.61	0.10
23	n-Hexadecane	AH	28.06	1600.3	nd	0.10
24	γ-Eudesmol	OS	28.92	1632.4	0.20	0.21
25	β-Eudesmol	OS	29.35	1651.2	0.15	0.16
26	α-Eudesmol	OS	29.43	1660.2	0.26	0.34
27	8-Heptadecene	AH	29.77	1682.5	nd	0.31
28	n-Heptadecane	AH	30.26	1700.1	0.35	2.80
29	Farnesyl alcohol	OS	30.87	1725.2	0.06	1.50
30	n-Octadecane	AH	32.39	1800.1	nd	0.28
31	1-Nonadecene	AH	33.90	1880.2	7.47	6.15
32	n-Nonadecane	AH	34.42	1900.6	16.57	14.19
33	n-Eicosane	AH	36.33	2000.3	2.18	2.38

34	10-Heneicosene	AH	38.00	2092.9	0.88	0.20
35	n-Heneicosane	AH	38.20	2100.4	15.78	8.53
36	n-Docosane	AH	39.96	2200.3	0.52	0.35
37	(Z)-9-Tricosene (Muscalure)	AH	41.50	2293.5	0.92	0.51
38	n-Tricosane	AH	41.64	2300.2	4.23	3.21
39	n-Tetracosane	AH	43.28	2400.2	0.20	0.20
40	n-Tetracosanol-1 (Lignocerol)	OA	44.73	2495.0	0.10	0.11
41	n-Pentacosane	AH	44.84	2502.6	0.10	0.20
42	n-Hexacosane	AH	44.84	2600.1	1.35	1.13

RT - retention time; min.
RI – retention index

	***Rosa alba* L.**	***Rosa damascena* Mill.**
Oxygenated monoterpenes (OM)	17.64	14.57
Sesquiterpenes hydrocarbons (SH)	0.21	0.74
Benzenoid compounds (BC)	11.83	29.22
Aliphatic hydrocarbons (AH)	50.949	38.47
Oxygenated aliphatics (OA)	1.09	0.25
Triterpenes (T)	0.43	nd
Pentacyclic triterpenoids (PT)	3.452	9.75
